# Integrated Community Case Management of Childhood Illness in Ethiopia: Implementation Strength and Quality of Care

**DOI:** 10.4269/ajtmh.13-0751

**Published:** 2014-08-06

**Authors:** Nathan P. Miller, Agbessi Amouzou, Mengistu Tafesse, Elizabeth Hazel, Hailemariam Legesse, Tedbabe Degefie, Cesar G. Victora, Robert E. Black, Jennifer Bryce

**Affiliations:** Institute for International Programs, Department of International Health, Johns Hopkins Bloomberg School of Public Health, Baltimore, Maryland; ABH Services, PLC, Addis Ababa, Ethiopia; United Nations Children's Fund (UNICEF) Ethiopia Country Office, Addis Ababa, Ethiopia; Postgraduate Program in Epidemiology, Federal University of Pelotas, Pelotas, Brazil

## Abstract

Ethiopia has scaled up integrated community case management of childhood illness (iCCM) in most regions. We assessed the strength of iCCM implementation and the quality of care provided by health extension workers (HEWs). Data collectors observed HEWs' consultations with sick children and carried out gold standard re-examinations. Nearly all HEWs received training and supervision, and essential commodities were available. HEWs provided correct case management for 64% of children. The proportions of children correctly managed for pneumonia, diarrhea, and malnutrition were 72%, 79%, and 59%, respectively. Only 34% of children with severe illness were correctly managed. Health posts saw an average of 16 sick children in the previous 1 month. These results show that iCCM can be implemented at scale and that community-based HEWs can correctly manage multiple illnesses. However, to increase the chances of impact on child mortality, management of severe illness and use of iCCM services must be improved.

## Introduction

Pneumonia, diarrhea, and malaria are among the leading causes of mortality in children younger than 5 years old.^1^ Effective therapies for these conditions exist,^2^ but children in poor rural communities often do not have access to formal healthcare,^3^,^4^ and coverage of these interventions remains low in many countries.^5^ Delivery of care through community health workers (CHWs) can increase coverage of specific treatments^6^–^8^ and lead to substantial reductions in child mortality.^9^–^13^ The World Health Organization (WHO) and the United Nations Children's Fund (UNICEF) recommend integrated community case management (iCCM) of pneumonia, diarrhea, and malaria.^14^ However, experience with large-scale implementation of iCCM remains limited. In 2010, just 12 countries in sub-Saharan Africa were implementing CCM of at least three illnesses, and only 6 sub-Saharan African countries were implementing CCM of at least three illnesses in at least 50% of the country's districts.^15^ Few rigorous assessments of iCCM implementation have been conducted, and the limited evidence on quality of care is mixed.^16^–^22^

As part of Ethiopia's Health Extension Program, over 30,000 female health extension workers (HEWs) have been trained for 1 year and deployed to communities throughout the country to provide preventive and curative health services. There are typically two HEWs assigned to a kebele (subdistrict) with a population of approximately 5,000 people. HEWs usually provide clinical care in health posts but may also provide care in the homes of community members.

After a national policy change supporting community-based treatment of childhood pneumonia by HEWs in early 2010, Ethiopia has scaled up iCCM provided by HEWs in most regions of the country. Antibiotic therapy (with cotrimoxazole) for pneumonia and zinc for treatment of diarrhea have been added to the pre-existing CCM (routine CCM) program, which included management of childhood diarrhea (with oral rehydration salts [ORS] only), malaria, malnutrition, measles, ear infection, and anemia. Other than the addition of antibiotics and zinc, the iCCM program provides a number of program supports, including a 6-day training for HEWs on iCCM, strengthened supervision, improved supply chain management for essential commodities, and enhanced monitoring and evaluation. Definitions of key terms are shown in Box 1.

The scale-up of iCCM was implemented in a phased manner in Oromia Region. This allowed for randomization of rural woredas (districts) in two zones into phase one intervention areas (iCCM) and phase two comparison areas (routine CCM) for an independent evaluation of the impact of iCCM. In addition to the present study, data for the evaluation came from baseline and endline household surveys to measure child mortality and coverage of treatments for common childhood illnesses.

This study was commissioned by UNICEF to assess whether iCCM implementation strength, quality of care, and use of services were sufficient to plausibly expect a substantial impact from the intervention on child mortality at the population level. We conducted a cross-sectional survey of health posts where HEWs work in intervention and comparison areas. The study had two main objectives: (1) measure and compare indicators of iCCM implementation strength in intervention areas and routine CCM implementation strength in comparison areas and (2) assess the quality of iCCM services provided to sick children in intervention areas. Quality of care was not assessed in comparison areas because the primary purpose of the study was to assess implementation strength and quality of care of iCCM; also, funding for data collection in comparison areas was limited.

This survey was the first to evaluate the scale-up of iCCM in Ethiopia and the first rigorous assessment of the quality of care provided by iCCM-trained HEWs. It was also one of the first studies to assess the quality of iCCM services in sub-Saharan Africa and the first study to assess integrated management of childhood pneumonia, diarrhea, malaria, and malnutrition by CHWs.

## Materials and Methods

### Study population.

The survey was conducted in rural areas of Jimma and West Hararghe Zones of Oromia Region. Oromia is Ethiopia's largest region, with approximately 30 million people.^23^ Eighty-three percent of the population lives in rural areas, and the primary occupation is subsistence agriculture.^24^ Malaria risk varies in the study areas, with about one-quarter of health posts in high-malaria risk areas, 25% with no malaria, and the rest with low malaria risk. The study was conducted just before the start of the rainy, high-malaria transmission season. Jimma and West Hararghe Zones have populations of approximately 2.9 and 2.1 million people, respectively.^25^

### Survey design and inclusion criteria.

We conducted a cross-sectional survey of rural health posts in the two study zones. The sample was stratified by iCCM intervention and comparison areas. Intervention areas received the iCCM program detailed above, whereas comparison areas continued with the pre-existing routine CCM program. Table 1 details the differences between the iCCM and the routine CCM programs. Systematic random sampling was used to select health posts within each stratum.

All HEWs present and providing case management services in selected health posts were included. Children had to meet the following inclusion criteria: (1) 2–59 months of age (children younger than 2 months of age were excluded because there are separate clinical guidelines for this age group that call for referral to a health facility for most cases; also, we expected an extremely small sample of children in this age group, which would not justify the extra expense of training data collectors on the algorithm for children younger than 2 months of age), (2) having at least one complaint consistent with an eligible iCCM illness, and (3) initial consultation for the current illness episode.

### Sample size.

We selected 104 health posts from intervention areas and 46 health posts from comparison areas. Proportions of the indicators were assumed to be 50% to give the most conservative sample size. Confidence was set at 95%. We adjusted for non-response of 5% for health posts, 5% for HEWs, and 10% for patients. A design effect of 1.3 was included to account for clustering of HEWs and patients in health posts. Assuming an average of 1.5 HEWs per health post, 104 health posts allowed for estimates of health post- and HEW-level indicators in intervention areas with a precision of ±10% points. Assuming two sick children per health post, primary patient-level indicators would have a precision of ±9% points. The smaller sample size in comparison areas allowed a precision of ±15% points for health post- and HEW-level indicators.

### Instruments and indicators.

The survey instruments and primary indicators of implementation strength and quality of care were adapted from the WHO Health Facility Survey tool,^26^ a survey of Health Surveillance Assistants in Malawi,^21^ and the CCM Global Indicators.^27^ Indicator definitions are presented in Supplemental Appendix 1.

### Data collection.

Data were collected in May and June of 2012, about 1 year after completion of training of HEWs in iCCM and the beginning of iCCM implementation in the intervention areas. Data collectors were health professionals who had worked as iCCM trainers or supervisors. Survey personnel were trained for 7 days, and all observers and re-examiners achieved at least 90% concordance with gold standard clinicians on three consecutive role play examinations. Data collectors were sent to woredas in which they did not normally work to avoid biasing the results.

### Assessment of program implementation strength.

In intervention and comparison areas, survey teams collected information on the strength of iCCM/routine CCM implementation. Data collectors inspected iCCM commodities, supplies, and job aids. Next, they conducted interviews with the HEWs to learn about training and supervision received as well as HEW characteristics. Finally, they recorded information on sick child consultations from patient registers.

### Assessment of quality of care.

The assessment of quality of care was conducted in intervention areas only. HEWs were notified of upcoming study visits and asked to mobilize caretakers to bring sick children to the health post on the day of the survey team visit. If fewer than two children presented at the health post within 1 hour of the health post opening, the team supervisor, along with an HEW or community volunteer, recruited sick children from nearby households. Eligible children received a consultation from an HEW while a data collector silently observed and recorded details of the HEW's assessment, classification, treatment, referral, and counseling. Next, the observer took the patient and caretaker away from the HEW and asked the caretaker to explain how they would administer any treatments prescribed for the child. Finally, the re-examiner performed a consultation with the patient and caretaker using a re-examination form that followed Ethiopia iCCM clinical guidelines.

### Data analysis.

Data were entered directly into tablet computers using the Open Data Kit (ODK)^28^ as the data capture software and stored in a Research Electronic Data Capture (REDCap) database.^29^ All analyses were carried out in Stata 12.^30^ Descriptive statistics comprising proportions or means were calculated for selected indicators. Standard errors and associated 95% confidence intervals for HEW- and patient-level variables were calculated using the Taylor linearization method to account for clustering within health posts.^31^ Indicators of correct classification or treatment were calculated by comparing the HEW's classification and treatment with the gold standard classification from the re-examination and the associated treatments/referral recommended in the iCCM clinical guidelines. We performed clinical pathways analyses stratified by children with severe and uncomplicated illness to identify where case management errors occurred during assessment, classification, treatment, and referral of sick children.^32^,^33^ The full research protocol and study instruments are available online.^34^

### Ethical considerations.

Ethical approval was obtained from the Institutional Review Boards (IRBs) of the Oromia Regional Health Bureau and the Johns Hopkins University Bloomberg School of Public Health. Informed oral consent was obtained from all HEWs and caretakers of sick children. Written consent from all participants would not have been possible because many of the participants were not literate. The IRBs approved the use of oral consent, and data collectors noted on a form whether oral consent was obtained from each participant.

## Results

One health post in the intervention areas was excluded, because it was closed indefinitely, giving 103 health posts in intervention areas and 46 health posts in comparison areas that were successfully surveyed. All HEWs encountered in health posts were included in the study, resulting in samples of 137 HEWs in intervention areas and 64 HEWs in comparison areas. In total, 257 children were included in intervention areas.

Table 2 shows the characteristics of the HEWs included in the sample. They were all women, and HEWs in both study arms had an average of about 4 years of experience as an HEW; 91% of HEWs in intervention areas and 86% of HEWs in comparison areas reported living in the same kebele in which they work. However, only 12% of HEWs in intervention areas and 6% of HEWs in comparison areas reported having lived in the kebele in which they work before beginning their initial HEW training, indicating that most HEWs were not selected from the communities in which they work.

Table 3 presents the characteristics of sick children in the sample. According to the gold standard classifications, diarrhea (66%), pneumonia (15%), malnutrition (13%), and ear infection (12%) were the most common illnesses. Few children presented with malaria (1%), measles (2%), or anemia (4%). Spontaneous consultations accounted for only 18% of the sample of children; 37% of children were mobilized by the HEWs, and active recruitment of sick children accounted for 45% of the sample.

### Program implementation strength.

Table 4 shows the proportion of HEWs that received training and the proportion of health posts that received supervision in intervention and comparison areas. Nearly all HEWs (98%, 95% confidence interval [95% CI] = 93–99) in the intervention areas received the iCCM training. HEWs in 87% (95% CI = 79–93) of health posts received at least one supervision visit related to iCCM in the previous 3 months, and 85% (95% CI = 77–91) of health posts received supervision that included clinical reinforcement (observation of consultations or register review with feedback). As expected, no HEWs in comparison areas had received the iCCM training. Only 43% (95% CI = 28–59) of health posts in comparison areas had been supervised on routine CCM in the previous 3 months, and 19% (95% CI = 9–34) of health posts received supervision with clinical reinforcement.

Table 5 presents the proportions of health posts with key iCCM/routine CCM commodities, supplies, and job aids on the day of data collection and health posts with no stockout of more than 7 consecutive days in the previous 3 months in intervention and comparison areas; 69% (95% CI = 59–78) of intervention health posts had all seven essential commodities for iCCM on the day of data collection. The proportion of health posts with individual items in stock ranged from 99% (cotrimoxazole) to 80% (ready-to-use therapeutic food). Only 4% (95% CI = 1–15) of health posts in comparison areas had all five essential commodities for routine CCM in stock. About one-half (52%, 95% CI = 42–61) of health posts in intervention areas and all health posts in comparison areas reported a stockout lasting longer than 7 consecutive days in the previous 3 months of at least one of the essential items. Just under one-half (46%, 95% CI = 36–56) of intervention health posts and no comparison health posts had all essential supplies and job aids in stock on the day of the visit. Nearly all indicators of implementation strength were significantly higher in intervention areas than comparison areas.

### Quality of care.

Table 6 presents key indicators of quality of care in intervention areas. HEWs completed an average of 9.2 of 11 key assessment tasks. A large majority of children (81%, 95% CI = 74–86) was assessed for the presence of cough, diarrhea, fever, and malnutrition. Fewer children (62%, 95% CI = 53–70) were assessed for all four general danger signs. Just over one-half of children (53%, 95% CI = 46–60) were correctly classified for all major iCCM illnesses.

Nearly two-thirds of children (64%, 95% CI = 57–71) were correctly managed for all major iCCM illnesses. HEWs correctly treated 72% (95% CI = 56–84) of children with pneumonia, 79% (95% CI = 72–85) of children with diarrhea, and 59% (95% CI = 40–76) of children with malnutrition. Sample sizes of children with malaria and measles were too small to draw meaningful conclusions about management of those illnesses. Only 6% (95% CI = 3–10) of children received an antibiotic when it was not indicated, and no children received an unnecessary antimalarial. Just 34% (95% CI = 22–50) of children with severe illness were correctly managed, and HEWs referred about one-half (54%, 95% CI = 41–67) of children needing referral to a health center. Among 13 children with severe illness who were not referred, 8 (62%, 95% C= 31–85) children received correct treatment for their illnesses (excluding the requirement to refer). Furthermore, few children (14%, 95% CI = 8–21) received the first dose of all needed treatments in the presence of the HEW. Only 18% (95% CI = 10–31) of children needing a vitamin A supplement received vitamin A, and 20% (95% CI = 9–39) of children needing deworming medication received mebendazole. Over three-quarters of caretakers (74%, 95% CI = 63–83) received a demonstration on how to administer all treatments by the HEW, and 83% (95% CI = 76–89) of caretakers correctly described how to give all treatments.

**Figure 1.**
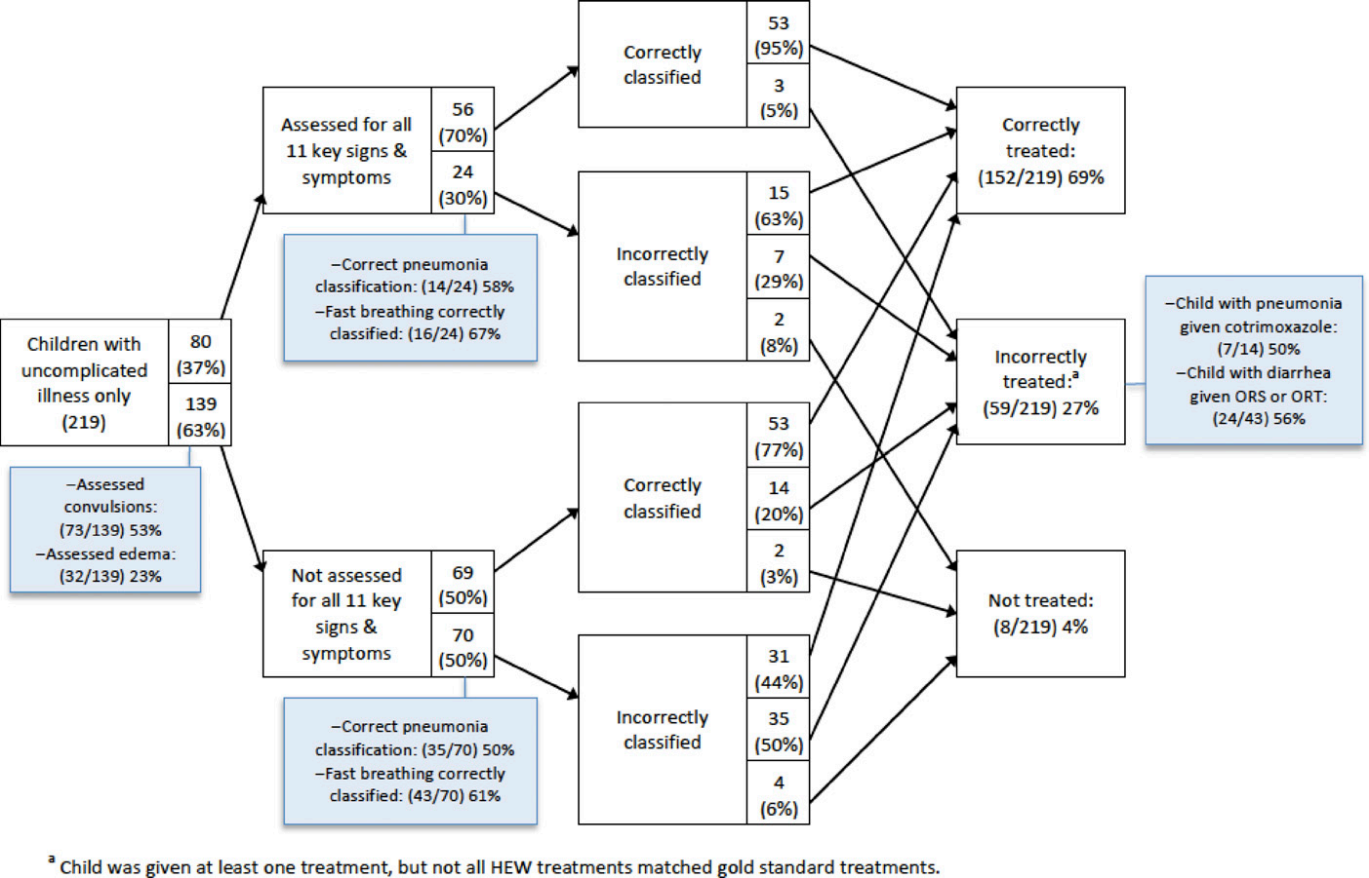
Clinical errors analysis for children with uncomplicated illnesses in intervention areas in Jimma and West Hararghe Zones, Oromia Region, Ethiopia. ORT = oral rehydration therapy.

**Figure 2.**
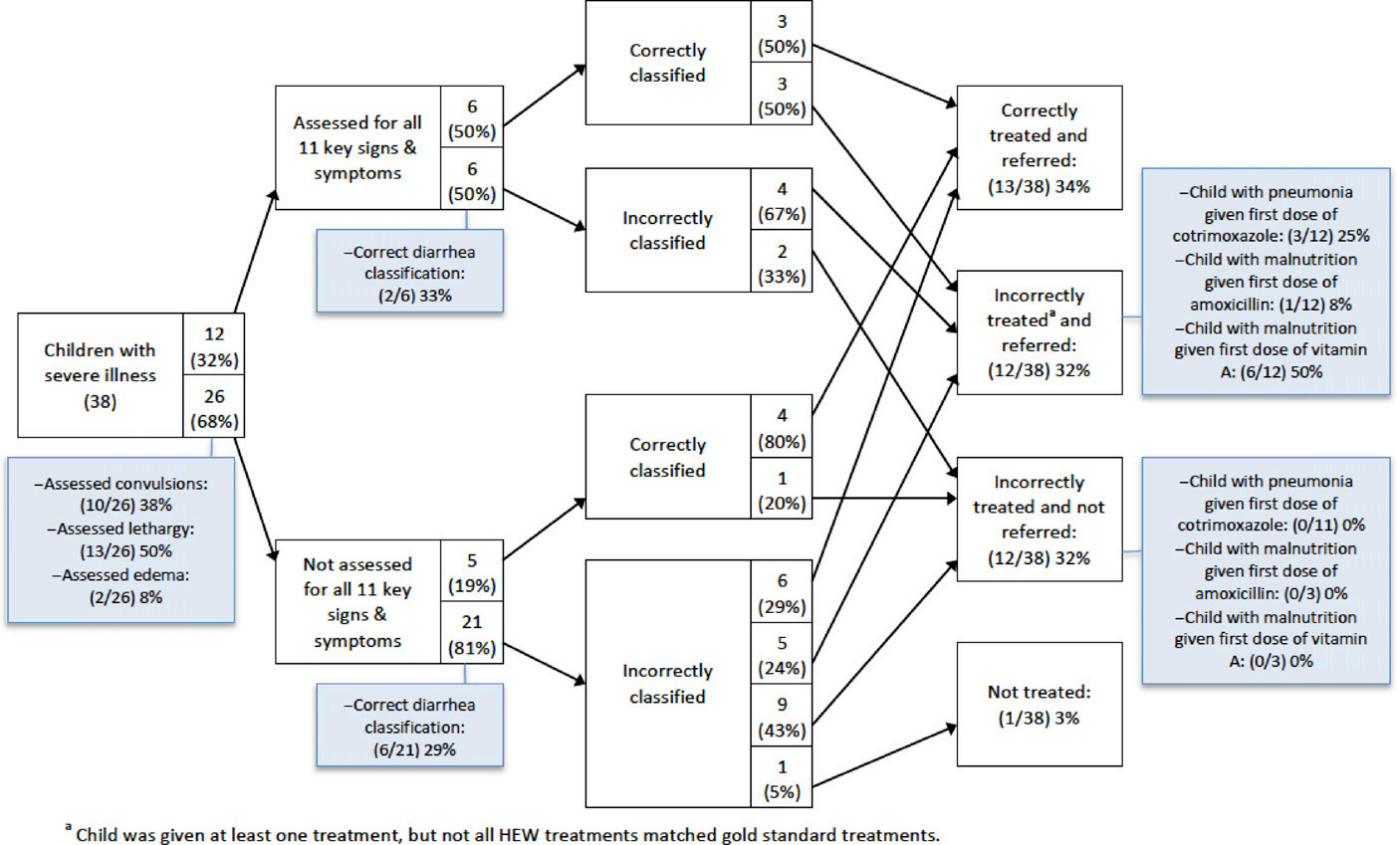
Clinical errors analysis for children with at least one severe illness in intervention areas in Jimma and West Hararghe Zones, Oromia Region, Ethiopia.

Figures 1 and 2 present analyses of clinical errors for children with uncomplicated illness only and children with at least one severe illness in intervention areas, respectively. Around one-third of children were assessed for all 11 selected signs and symptoms of iCCM illnesses. The most common assessment errors were failures to assess convulsions, edema, and lethargy. Incorrect classification of pneumonia was the most common classification error for children with uncomplicated illness, partially because of incorrect assessment of fast breathing. The most common treatment errors were failure to give cotrimoxazole to children with pneumonia and failure to give ORS for diarrhea, although these items were in stock. Misclassification was common among children with severe illness, and incorrect treatment was common regardless of whether children were correctly classified. The most common treatment errors for children with severe illness were failure to give the first dose of cotrimoxazole for pneumonia, failure to give the first doses of amoxicillin and vitamin A for severe complicated malnutrition, and not referring children to health centers when it was required.

### Use and service provision.

Table 7 presents the mean number of sick child consultations per health post in the previous 1 month and selected indicators of service provision in intervention and comparison areas. Intervention health posts saw an average of 16 (95% CI = 13–19) sick children in the previous 1 month (range = 0–95) compared with 5 (95% CI = 2–8) sick children seen in comparison health posts (range = 0–32). Virtually no children under 2 months of age were seen in intervention or comparison areas.

When asked about their activities the day before the interview, HEWs in intervention areas reported spending an average of 6.1 hours on work tasks. They spent an average of about 4 hours providing or offering clinical services in the health post, about 30 minutes providing clinical services in the community, and nearly 1 hour carrying out community mobilization/education activities. HEWs in comparison areas spent less time in health posts and more time in the community. Intervention and comparison health posts were reportedly open and offering clinical services for an average of about 20 hours in the previous week.

## Discussion

Several countries and international agencies are pushing for rapid scale-up of iCCM, despite a dearth of evidence on best practices for implementation, quality of care, and impact. The scale-up of iCCM in Ethiopia is the most ambitious implementation of the strategy in sub-Saharan Africa to date. The country also has the advantage of a large literate, relatively well-trained, and salaried cadre of HEWs. Thus, it is important to take note of the lessons from the implementation of iCCM in Ethiopia for both improvement of the Ethiopia program and implementation in other countries as they prepare to scale up iCCM.

iCCM has largely been implemented as planned in the study areas. Nearly all HEWs were trained in iCCM, and 87% had received supervision in iCCM in the previous 3 months. Nearly 70% of health posts had all essential commodities for iCCM.

HEWs in iCCM intervention areas performed most basic assessment tasks and correctly managed nearly two-thirds of all children, with minimal overprescription of drugs. Comparisons of our results with other studies with similar assessment methods show that adherence to clinical guidelines seems to be at least as high for HEWs as for health workers at hospitals and health centers in Ethiopia.^35^ Quality of care provided by HEWs also compares favorably with performance in management of multiple childhood illnesses by CHWs in Malawi,^21^ Kenya,^17^ and Papua New Guinea.^16^ A recent review of studies of CCM of pneumonia in sub-Saharan Africa concluded that CHWs had difficulties with counting children's respiratory rate, correct classification and treatment of pneumonia, and overuse of antibiotics.^22^ We found that HEWs counted respiratory rates more accurately, correctly treated a higher proportion of children with pneumonia, and overprescribed antibiotics less than CHWs in the previous studies.

Despite these achievements, only about one-third of children with severe illness were correctly managed, which is consistent with prior research.^17^,^36^ An important reason for this result was that only about one-half of children needing referral to a health facility were referred by the HEW. Other studies have shown that many referred children never reached the referral facility or caretakers complied with referral only after substantial delays.^37^,^38^ Lack of transportation, cost of transportation, fees for care at referral facilities, and responsibilities at home can all discourage compliance with referral.^39^,^40^ The failure of HEWs to refer children with severe illness seen in this study may have been attributable to the fact that the HEWs did not believe the caretakers would comply, and therefore, they preferred to treat the children in the health post.

Studies from Pakistan have shown that CHWs can diagnose and treat severe pneumonia and that CCM of severe pneumonia can reduce treatment failures.^41^,^42^ However, more evidence is needed, especially from sub-Saharan Africa. Research is also needed on the reasons for mismanagement of children with severe illness and whether health outcomes can most feasibly and effectively be improved through enhanced training of CHWs on recognition of severe illness and danger signs, facilitation of transportation for referral, or enabling CHWs to treat children with severe illness in the community. Although mismanagement of children with severe illness is a serious concern, it should be noted that, even if referral rates are low, iCCM can still contribute to substantial reductions in mortality through the early management of illnesses, thus preventing children from becoming severely ill in the first place.

Few children accessed care from HEWs, and virtually no children under 2 months of age, the age group with the highest risk of mortality, were seen. Box 2 presents an estimation of the gap between expected and actual sick child consultations in intervention areas. Using the average number of consultations in the previous 1 month and the estimated number of children under 5 years old in the health post catchment area at surveyed health posts, there were 0.26 consultations at health posts per child per year. If we assume that patients should seek care from HEWs for 25–50% of illness episodes (a conservative estimate considering that HEWs are meant to be the primary source of care in the public health system and that care-seeking from other sources is low^43^), only 16–38% of expected consultations from HEWs were actually seen. More encouragingly, significantly higher use in intervention areas than comparison areas suggests that use may have increased where the iCCM program was introduced and services improved. Furthermore, there was a large range in the number of consultations in the previous 1 month, with several health posts registering two to three times the average. More research is needed to understand why some health posts have much higher use than others.

Low use of child health services is a common challenge. It was seen in the multicountry evaluation of integrated management of childhood illness (IMCI), where improvements in quality of care were mitigated by low levels of care-seeking at health facilities and a failure to reach sick young infants (younger than 2 months old).^44^–^46^ Although CCM is meant to substantially increase access to care, uptake of community-based services is often disappointing. A global review of data on sources of care for sick children from Demographic and Health Surveys (DHSs) and Multiple Indicator Cluster Surveys (MICS) in 42 countries in sub-Saharan Africa and Asia found that CHWs generally were not an important source of care.^47^

To earn the trust of the communities and attain greater uptake of services, program implementers must ensure consistent availability of health workers and essential commodities. Demand creation activities and increased active case detection by HEWs are also urgently needed. The experience of recruiting children for this survey suggests that there are large numbers of children with easy access to HEWs who fail to use their services despite being seriously ill. The Ethiopian Government's launch of the “Health Development Army,” for which up to 3 million community members will be mobilized nationally to serve as community health volunteers, presents a promising platform through which to carry out large-scale demand creation.^48^ However, a thorough evaluation of this new initiative is needed.

This study has a number of limitations. First, small sample sizes of children with malaria and measles precluded assessment of management of those illnesses. Second, HEWs may have performed better under observation than they normally would have performed.^49^,^50^ Third, informing HEWs of survey visits may have allowed them to prepare for the visits, which would have further biased the results positively. Fourth, recruitment and mobilization of sick children may have provided a sample of patients that was different from spontaneous patients, although concerns that we would obtain few children with severe illness proved unfounded. If these children were different from the children that the HEWs normally would see, it could have biased the results. Fifth, some information was based on self-report by HEWs, which may be biased. Sixth, iCCM program implementers knew which woredas were selected as intervention areas; therefore, extra effort may have been given to these areas, and implementation may have been stronger than in other areas of the country. Seventh, because the HEWs are a relatively well-trained and well-remunerated cadre of health workers, the results on HEW performance may not be generalizable to CHWs with a lower skill level and fewer incentives. These limitations may have resulted in observed levels of implementation strength and quality of care that were higher than they would have been under normal circumstances.

Scaling up iCCM at a national or regional level poses considerable challenges. The scale of the task of deploying CHWs and ensuring adequate training, sustained supervision, and availability of essential commodities can be daunting, but this challenge is well-recognized. The additional challenge of ensuring community demand for iCCM services is equally important but may be overlooked. Without concerted efforts to promote use of services, millions of dollars spent on implementation of iCCM may have little impact on child mortality.

## Supplementary Material

Supplemental Appendix.

## Figures and Tables

**Box 1:** Definitions of key terms

Key terms
Correct classification: All HEW classifications matched gold standard classifications.
Correct treatment: All HEW treatments matched gold standard treatments, including correct dose, duration, and frequency.
Coverage of therapeutic interventions: The proportion of children under 5 years old with an illness who received correct treatment of that illness.
Eligible iCCM illness: Lethargy or unconsciousness, convulsions, not eating or drinking, fever/malaria, cough, fast/difficulty breathing, diarrhea, vomiting, ear problem, signs/history of measles, malnutrition, feeding problems, or anemia.
General danger signs: Not able to drink/breastfeed, vomits everything, had convulsions, and lethargy.
Implementation strength: This term refers to the quantity of effective program activities carried out to reduce child mortality. These activities include training, supportive supervision, and continued availability of essential iCCM commodities.
iCCM : In general, iCCM refers to the concurrent management of more than one common childhood illness. iCCM in Ethiopia (implemented in study intervention areas) is integrated management by an HEW at the community level of all of the following childhood illnesses: pneumonia, diarrhea (ORS and zinc), malaria, malnutrition, measles, anemia, and ear infection.
Major iCCM illnesses: Pneumonia, diarrhea, malaria, measles, malnutrition, and danger signs.
Quality of care: We assessed quality based on whether HEWs correctly assessed, classified, treated, and referred children with iCCM illnesses and provided counseling to caretakers based on Ethiopia iCCM clinical guidelines.
Routine CCM: The routine CCM program in Ethiopia (implemented in study comparison areas) includes CCM of diarrhea (ORS only), malaria, malnutrition, measles, ear infection, and anemia. Pneumonia cases are referred to health centers.
Severe illness: A child was considered to have a severe illness if the child had any of the following signs: any general danger sign (not able to drink/breastfeed, vomits everything, convulsions, lethargic, or unconscious), severe pneumonia, diarrhea with severe dehydration, severe persistent diarrhea, persistent diarrhea, dysentery, very severe febrile disease, severe complicated measles, severe complicated malnutrition, or severe anemia.
Supervision with clinical reinforcement: Supervision with observation of patient consultations or register review.
Eleven key assessment tasks: Checked whether child is able to drink/breastfeed, checked whether child vomits everything, checked whether child has had convulsions, checked whether child has lethargy, checked for cough or fast/difficult breathing, checked for diarrhea, checked for fever, checked for edema, checked for low MUAC (≥ 6 months) or visible severe wasting (< 6 months), checked for palmar pallor, and checked child's vaccination status.

**Table 1:** Comparison of case management guidelines and program inputs for the Ethiopia iCCM program and routine CCM

	ICCM (intervention areas)	Routine CCM (comparison areas)
Management of iCCM illnesses for children 2–59 months		
Pneumonia	Cotrimoxazole	Referral to health center
Severe pneumonia	Pre-referral treatment with cotrimoxazole; referral to health center	Referral to health center
Diarrhea (some dehydration, no dehydration)	ORS/ORT; zinc	ORS/ORT
Severe diarrhea (severe dehydration, persistent diarrhea, severe persistent diarrhea, dysentery)	ORS; vitamin A (for persistent and severe persistent diarrhea only); referral to health center	ORS; vitamin A (for persistent and severe persistent diarrhea only); referral to health center
Malaria	Antimalarial	Antimalarial
Severe febrile disease	Pre-referral treatment with cotrimoxazole; referral to health center	Referral to health center
Uncomplicated malnutrition	RUTF or supplementary feeding program	RUTF or supplementary feeding program
Severe complicated malnutrition	Pre-referral treatment with amoxicillin and vitamin A; referral to health center	Pre-referral treatment with amoxicillin and vitamin A; referral to health center
Measles	Vitamin A	Vitamin A
Severe complicated measles	Vitamin A; cotrimoxazole; tetracycline eye ointment (optional); referral to health center	Vitamin A; tetracycline eye ointment (optional); referral to health center
Measles with eye or mouth complications	Vitamin A; tetracycline eye ointment (optional); gentian violet (optional)	Vitamin A; tetracycline eye ointment (optional); gentian violet (optional)
Acute ear infection	Paracetamol; referral to health center	Paracetamol; referral to health center
Anemia	Referral to health center	Referral to health center
Program inputs		
Training	6 days of training on iCCM	No additional training
Supervision	Standardized supportive supervision on iCCM supported by partner NGOs plus standard government supervision; biannual PRCM meetings	Standard government supervision
Supply of commodities	Support for purchase and supply of drugs and other commodities by UNICEF and partners; provision of iCCM registers, iCCM chart booklets, timers, and other supplies	Standard government commodity supply chain system; no additional supplies or job aids
Monitoring and evaluation	Enhanced data collection during supervisions and PRCM meetings; data management support by UNICEF	Standard government monitoring and evaluation

NGO = non-governmental organization; ORT = oral rehydration salts; PRCM = performance review and clinical mentoring; RUTF = ready-to-use therapeutic food.

**Table 2:** Distribution of HEWs by selected characteristics in intervention (*N* = 137) and comparison (*N* = 64) areas in Jimma and West Hararghe Zones, Oromia Region, Ethiopia in 2012

Characteristic	Intervention areas		Comparison areas	
	*n*	Percent	*n*	Percent
Age, years				
18–20	13	9.5	9	14.1
21–23	83	60.6	35	54.7
24–26	35	25.6	18	28.1
27–29	4	2.9	1	1.6
30–32	2	1.5	1	1.6
Marital status				
Married	80	58.4	39	60.9
Single	56	40.9	25	39.1
Separated/divorced	1	0.7	0	0.0
HEW lives in the same kebele as health post	125	91.2	55	85.9
HEW lived in the kebele 1 year before completing basic HEW training	16	11.7	4	6.3

**Table 3:** Distribution of sick children by selected characteristics in intervention areas in Jimma and West Hararghe Zones, Oromia Region, Ethiopia in 2012 (*N* = 257)

Characteristic	*n*	Percent
Age, months		
2–11	94	36.6
12–23	92	35.8
24–35	39	15.2
36–47	22	8.6
48–59	10	3.9
Sex		
Male	129	50.2
Female	128	49.8
Gold standard disease classifications		
Pneumonia	39	15.2
Diarrhea	169	65.8
Malaria/severe febrile disease	3	1.2
Measles	5	2.0
Malnutrition	32	12.5
Ear infection	30	11.7
Anemia	11	4.3
Severe illness	38	14.8
Needs referral*	63	24.5
Method of recruitment		
Spontaneous	45	17.5
Mobilized by HEWs	96	37.4
Recruited by survey team	116	45.1

* In addition to severe illnesses, acute ear infection and anemia require referral.

**Table 4:** Selected indicators of training and supervision in intervention and comparison areas in Jimma and West Hararghe Zones, Oromia Region, Ethiopia in 2012

Indicator	Intervention areas		Comparison areas		*P* value†
	N*	Percent (95% CI)	*N*	Percent (95% CI)	
HEW trained in iCCM	137	97.8 (93.3–99.3)	64‡	0.0	< 0.001
Health post received supervision on iCCM/routine CCM in the previous 3 months	100§	87.0 (78.8–92.9)	42¶	42.9 (27.7–59.0)	< 0.001
Health post received supervision on iCCM/routine CCM that included register review or observation of consultations in the previous 3 months	100§	85.0 (76.5–91.4)	42¶	19.1 (8.6–34.1)	< 0.001
HEW received instruction in iCCM/routine CCM clinical practice at a health center in the previous 3 months	137	57.7 (48.8–66.0)	64	7.8 (3.1–18.3)	< 0.001

* Number of HEWs or health posts eligible for indicator.

† Two-sample binomial test of difference in proportions between intervention and comparison areas.

‡ HEWs in comparison areas were not expected to be trained in iCCM, and therefore, this result confirms that there was little to no spillover of iCCM training to HEWs outside of the intervention areas.

§ Three health posts were excluded, because HEWs reported not being present for the majority of the previous 3 months.

¶ Four health posts were excluded, because HEWs reported not being present for the majority of the previous 3 months.

**Table 5:** Availability of essential iCCM/routine CCM commodities, supplies, and job aids on the day of data collection and no stockout of essential commodities in the 3 months preceding the survey in intervention (*N* = 103) and comparison (*N* = 46) areas in Jimma and West Hararghe Zones, Oromia Region, Ethiopia in 2012

Item	Available on day of data collection					No stockout > 7 days in the last 3 months				
	Intervention areas		Comparison areas		*P* value*	Intervention areas		Comparison areas		*P* value
	*n*	Percent (95% CI)	*n*	Percent (95% CI)		*n*	Percent (95% CI)	*n*	Percent (95% CI)	
All essential commodities for iCCM/routine CCM†	71	68.9 (59.1–77.7)	2	4.4 (0.5–14.8)	< 0.001	53	51.5 (41.4–61.4)	0	0.0	< 0.001
Cotrimoxazole‡	102	99.0 (94.7–100)	1	2.2	< 0.001	102	99.0 (94.7–100)	2	4.4 (0.5–14.8)	< 0.001
ORS	100	97.1 (91.7–99.4)	28	60.9 (45.4–74.9)	< 0.001	93	90.3 (82.9–95.2)	28	60.9 (45.4–74.9)	< 0.001
Zinc‡	99	96.1 (90.4–98.9)	0	0.0	< 0.001	83	80.6 (71.6–87.7)	0	0.0	< 0.001
ACT	91	88.4 (80.5–93.8)	23	50.0 (34.9–65.1)	< 0.001	90	87.4 (79.4–93.1)	26	56.5 (41.1–71.1)	< 0.001
Chloroquine	92	89.3 (81.7–94.5)	17	37.0 (23.2–52.5)	< 0.001	91	88.4 (80.5–93.8)	18	39.1 (25.1–54.6)	< 0.001
RUTF	82	79.6 (70.5–86.9)	16	34.8 (21.4–50.2)	< 0.001	80	77.7 (68.4–85.3)	14	30.4 (17.7–45.8)	< 0.001
RDT	92	89.3 (81.7–94.5)	29	63.0 (47.5–76.8)	< 0.001	91	88.4 (80.5–93.8)	29	63.0 (47.5–76.8)	< 0.001
All essential supplies and job aids for iCCM/routine CCM§	47	45.6 (35.8–55.7)	0	0.0	< 0.001					
Functional timer	94	91.3 (84.1–95.9)	5	10.9 (3.6–23.6)	< 0.001					
Thermometer	82	79.6 (70.5–86.9)	30	65.2 (49.8–78.6)	0.06					
Weighing scale	79	76.7 (67.3–84.5)	37	80.4 (66.1–90.6)	0.612					
MUAC tape	102	99.0 (94.7–100)	41	89.1 (76.4–96.4)	0.005					
Clean water	74	71.8 (63.0–80.7)	3	6.5 (1.4–17.9)	< 0.001					
Supplies for ORS¶	77	74.8 (65.2–82.8)	0	0.0	< 0.001					
Chart booklet with clinical guidelines	103	100	0	0.0	< 0.001					
Sick child register	103	100	7	15.2 (6.3–28.9)	< 0.001					

ACT = artimisinin-based combination therapy; MUAC = middle upper arm circumference; RDT = rapid diagnostic test (malaria).

* Two-sample binomial test of difference in proportions between intervention and comparison areas.

† Intervention areas: cotrimoxazole, ORS, zinc, ACT, chloroquine, RUTF, and RDT. Comparison areas: ORS, ACT, chloroquine, RUTF, and RDT.

‡ Cotrimoxazole and zinc were not part of the routine CCM program, and therefore, comparison health posts are not expected to have these drugs available.

§ Intervention areas: timer, thermometer, weighing scale, MUAC, clean water, supplies for ORS, iCCM chart booklet, and sick child register. Comparison areas: timer, thermometer, weighing scale, MUAC, clean water, supplies for ORS, and sick child register.

¶ Cup and spoon.

**Table 6:** Selected indicators of quality of case management by HEWs in intervention areas in Jimma and West Hararghe Zones, Oromia Region, Ethiopia in 2012

Indicator	N*	Percent	95% CI
Assessment			
Child assessed for four general danger signs	257	61.9	52.5–70.4
Child checked for presence of cough, diarrhea, fever, and malnutrition	257	80.5	73.6–86.0
Child with cough or difficult breathing assessed for fast breathing by counting of respiratory rate	148	93.2	85.8–96.9
Child's vaccination status checked (children under 12 months)	94	97.9	91.7–99.5
Child's respiratory rate counted by the HEW within ± five breaths of the gold standard	130	70.0	60.8–77.8
Classification			
Child correctly classified for all major iCCM illnesses	257	52.9	45.6–60.1
Child not up to date on immunizations classified as not up to date	77	36.4	26.1–48.1
Treatment and referral			
Child correctly treated† and/or referred for all major iCCM illnesses	257	64.2	57.4–70.5
Child with pneumonia correctly treated for pneumonia	39	71.8	55.8–83.7
Child with diarrhea correctly treated for diarrhea	169	79.3	71.5–85.4
Child with malnutrition correctly treated for malnutrition	32	59.4	40.4–76.0
Child with malaria correctly treated for malaria	3	66.7	2.0–100
Child with measles correctly treated for measles	5	20.0	0.3–94.9
Child with severe illness correctly treated and/or referred	38	34.2	21.5–49.7
Child needing referral correctly referred	63	54.0	40.7–66.7
Child received first dose of all needed treatments in the presence of the HEW‡	163	13.5	8.4–21.0
Child needing vitamin A supplementation received vitamin A	66	18.2	9.9–31.0
Child needing mebendazole received mebendazole	30	20.0	9.0–38.8
Child received an unnecessary antibiotic	257	5.5	3.0–9.7
Child received an unnecessary antimalarial	257	0.0	–
Counseling			
Caretaker received demonstration of how to administer all treatments by the HEW‡	160	74.4	63.4–82.9
Caretaker correctly described how to give all treatments‡	156	83.3	75.5–89.0
Caretaker advised to give extra fluids and continued feeding for diarrhea§	140	85.0	77.7–90.2
Caretaker advised to return immediately if the child cannot drink/breastfeed or becomes sicker§	213	36.2	27.3–46.0
Caretaker advised on when to return for follow-up§	213	93.4	88.0–96.5

* Number of children eligible for task.

† Includes prescription with correct dose, duration, and frequency.

‡ Includes cotrimoxazole, ORS, zinc, vitamin A, ACT, chloroquine, and amoxicillin and excludes children who were referred.

§ Excludes children who were referred.

**Table 7:** Mean number of sick child consultations per health post and selected indicators of service provision in intervention and comparison areas in Jimma and West Hararghe Zones, Oromia Region, Ethiopia in 2012

Indicator	Intervention areas		Comparison areas	
	Mean (95% CI)	Range	Mean (95% CI)	Range
Sick child consultations in previous 1 month				
Total	16.0 (13.2–18.8)	0–95	5.0 (2.3–7.7)	0–32
0 to < 2 months	0.3 (0.1–0.5)	0–9	0.03 (0.0–0.1)	0–1
2–59 months	15.7 (13.0–18.4)	0–94	5.0 (2.4–7.6)	0–31
Female	8.0 (6.6–9.5)	0–40	2.3 (0.7–3.9)	0–19
Male	7.9 (6.4–9.4)	0–57	2.4 (1.2–3.7)	0–13
Unspecified	0.1 (0.0–0.1)	0–4	0.3 (0.0–0.7)	0–6
Hours health post was open in previous 1 week	23.3 (21.0–25.5)	0–40	20.2 (17.0–23.5)	0–40
Hours spent by the HEW in the previous 1 day				
Providing clinical services in the health post	4.0 (3.5–4.5)	0–10	1.8 (1.1–2.5)	0–8
Providing clinical services in the community	0.5 (0.3–0.6)	0–7.5	0.8 (0.3–1.2)	0–5
Community education/mobilization; disease prevention	0.9 (0.6–1.1)	0–8	1.5 (0.9–2.2)	0–8
Other health-related activities	0.8 (0.5–1.1)	0–8	1.4 (0.7–2.1)	0–8
Other non–health-related activities	0.2 (0.1–0.4)	0–8	0.4 (0.1–0.8)	0–7
Travel outside kebele	0.7 (0.3–1.2)	0–12	0.5 (0.1–0.9)	0–8
Total work-related activities	6.1 (5.6–6.6)	0–11	5.5 (4.9–6.2)	0–9

**Box 2:** Estimation of the gap between expected and actual iCCM consultations in intervention health posts in Jimma and West Hararghe Zones, Oromia Region, Ethiopia in 2012

To illustrate the gap between expected and actual rates of use of iCCM services in intervention areas we conducted an estimation exercise using the following assumptions
There were on average 744 children under 5 years old per health post in intervention health posts in Jimma and West Hararghe according to statistics available in the health posts.
On average an under 5-year-old child is expected to have 0.28 episodes of pneumonia (based on estimated incidence among children 0–4 years old in Ethiopia in 2010) 51 3.3 episodes of diarrhea (based on estimated incidence among children under 5 years old in the WHO AFRO region in 2010) 52 and 0.17 episodes of malaria per year (based on incidence among children under 10 years old in Jimma Zone Oromia Region Ethiopia from 2008 to 2010).^53^ Thus, the average total number of episodes of these three illnesses in 1 year for one child would be 3.75. For a 1-year period, this would equal a total of 2,790 episodes per health post catchment area (excluding children with malnutrition and other illnesses included in the iCCM program).
If we assume that caretakers should seek care for 25–50% of those episodes from health posts/HEWs (others may seek care from other appropriate sources*) then we would expect 698–1,395 consultations for three illnesses per health post per year.
The current number of iCCM consultations per intervention health post per year based on use in 1 month before data collection is 192.
With 192 consultations per year for 744 children there are only 0.26 consultations per child per year at health posts.
Using the assumptions above there is a gap of 506–1,203 consultations per health post per year. In other words only 14–28% of expected consultations with HEWs are being seen.

* The iCCM evaluation baseline survey in Jimma and West Hararghe in 2011 found that 12.2%, 7.1%, and 11.6% of children with pneumonia, diarrhea, and fever, respectively, sought care at a public health facility other than HEW/health post.
