# Supplementary material for: Integrated Community Case Management of Childhood Illness in Ethiopia: Implementation Strength and Quality of Care
Source: Am J Trop Med Hyg. 2014 Aug 6;91(2):424–34. doi: 10.4269/ajtmh.13-0751 (PMC4125273; doi:10.4269/ajtmh.13-0751)
Supplement: Supplementary file 1 [file SD5.pdf]

Supplemental Appendix 1

Names, definitions, and measurement methods for primary indicators measured in the implementation snapshot and quality of care assessment in Jimma and West Hararghe Zones, Oromia Region, Ethiopia in 2012

| Indicator name | Indicator definition | Numerator | Denominator | Measurement method |
|---|---|---|---|---|
| HEW trained in iCCM | Proportion of HEWs who received the standardized iCCM training in February of 2011 or later | Number of HEWs who received the standardized iCCM training in February of 2011 or later | Number of HEWs | HEW interview |
| Availability of iCCM commodities | Proportion of health posts with all essential iCCM commodities in stock on the day of data collection | Number of health posts with all essential iCCM commodities in stock on the day of data collection | Number of health posts | Health post inspection |
| Continued medicine and diagnostic availability | Proportion of health posts with no stockout of any essential iCCM commodities lasting longer than 7 consecutive days in the previous 3 months | Number of health posts with no stockout of any essential iCCM commodities lasting longer than 7 consecutive days in the previous 3 months | Number of health posts | Health post inspection |
| Availability of iCCM supplies and job aids | Proportion of health posts with all essential supplies and job aids available on the day of data collection | Number of health posts with all essential supplies and job aids available on the day of data collection | Number of health posts | Health post inspection |
| ICCM supervision | Proportion of health posts that received at least one supervisory contact related to iCCM in the previous 3 months | Number of health posts that received at least one supervisory contact related to iCCM in the previous 3 months | Number of health posts | HEW interview |
| ICCM supervision with clinical reinforcement | Proportion of health posts that received at least one supervisory contact during the previous 3 months during which a sick child consultation was observed or the iCCM registration book was reviewed | Number of health posts that received at least one supervisory contact during the previous 3 months during which a sick child consultation was observed or the iCCM registration book was reviewed | Number of health posts | HEW interview |
| Health center visits with iCCM instruction | Proportion of HEWs who visited a health center and were instructed in iCCM clinical practice in the previous 3 months | Number of HEWs who visited a health center and were instructed in iCCM clinical practice in the previous 3 months | Number of HEWs | HEW interview |
| Use of iCCM services | Mean number of sick child consultations for children under 5 years old in the previous 1 month in surveyed health posts | Number of sick child consultations for children under 5 years old in the previous 1 month in surveyed health posts | Number of health posts | Register review |
| Assessment of general danger signs | Proportion of children who were assessed for all four general danger signs | Number of children who were assessed for all four general danger signs | Number of children | Observation of HEW consultation |
| Assessment of cough, diarrhea, fever, and malnutrition | Proportion of children who were assessed for presence of cough or fast/difficult breathing, diarrhea, fever, and malnutrition | Number of children who were assessed for presence of cough or fast/difficult breathing, diarrhea, fever, and malnutrition | Number of children | Observation of HEW consultation |
| Index of integrated assessment | Arithmetic mean of 11 key assessment tasks | Number of key assessment tasks performed for each child | Number of children | Observation of HEW consultation |
| Assessment of respiratory rate | Proportion of children with cough or difficult breathing who were assessed for fast breathing by counting of respiratory rate | Number of children with cough or difficult breathing who were assessed for fast breathing by counting of respiratory rate | Number of children with cough or difficult breathing | Observation of HEW consultation and re-examination |
| Respiratory rate correctly assessed | Proportion of children whose respiratory rate counted by the HEW was within ± five breaths of the gold standard | Number of children whose respiratory rate counted by the HEW was within ± five breaths of the gold standard | Number of children whose respiratory rate was counted by the re-examiner | Observation of HEW consultation and re-examination |
| Assessment of vaccination status | Proportion of children who did not need referral who had their vaccination status assessed by the HEW | Number of children who did not need referral who had their vaccination status assessed by the HEW | Number of children who did not need referral | Observation of HEW consultation and re-examination |

*(continued)*

Supplemental Appendix 1
Continued

| Indicator name | Indicator definition | Numerator | Denominator | Measurement method |
|---|---|---|---|---|
| Classification of iCCM illnesses | Proportion of children who were correctly classified for all major iCCM illnesses | Number of children who were correctly classified for all major iCCM illnesses | Number of children | Observation of HEW consultation and re-examination |
| Classification of immunization status | Proportion of children under 24 months of age not up to date on immunizations who were classified as not up to date on immunizations | Number of children under 24 months of age not up to date on immunizations who were classified as not up to date on immunizations | Number of children under 24 months of age not up to date on immunizations | Observation of HEW consultation and re-examination |
| Management of iCCM illnesses | Proportion of children who were correctly treated/referred for all major iCCM illnesses | Number of children who were correctly treated/ referred for all major iCCM illnesses | Number of children | Observation of HEW consultation and re-examination |
| Treatment of pneumonia | Proportion of children with pneumonia who were correctly treated for pneumonia | Number of children with pneumonia who were correctly treated for pneumonia | Number of children with pneumonia | Observation of HEW consultation and re-examination |
| Treatment of diarrhea | Proportion of children with diarrhea who were correctly treated for diarrhea | Number of children with diarrhea who were correctly treated for diarrhea | Number of children with diarrhea | Observation of HEW consultation and re-examination |
| Treatment of malaria | Proportion of children with malaria who were correctly treated for malaria | Number of children with malaria who were correctly treated for malaria | Number of children with malaria | Observation of HEW consultation and re-examination |
| Treatment of malnutrition | Proportion of children with malnutrition who were correctly treated for malnutrition | Number of children with malnutrition who were correctly treated for malnutrition | Number of children with malnutrition | Observation of HEW consultation and re-examination |
| Treatment of measles | Proportion of children with measles who were correctly treated for measles | Number of children with measles who were correctly treated for measles | Number of children with measles | Observation of HEW consultation and re-examination |
| Management of severe illness | Proportion of children with severe illness were correctly treated/referred | Number of children with severe illness were correctly treated/referred | Number of children with severe illness | Observation of HEW consultation and re-examination |
| Referral | Proportion of children needing referral who received referral | Number of children needing referral who received referral | Number of children needing referral | Observation of HEW consultation and re-examination |
| First dose | Proportion of children who did not need referral and needed treatment who received the first dose of all needed treatments in presence of the HEW | Number of children who did not need referral and needed treatment who received the first dose of all needed treatments in presence of the HEW | Number of children who did not need referral and needed treatment | Observation of HEW consultation and re-examination |
| Vitamin A supplementation | Proportion of children 6 months or older who needed vitamin A supplementation who received vitamin A | Number of children 6 months or older who needed vitamin A supplementation who received vitamin A | Number of children 6 months or older who needed vitamin A supplementation | Observation of HEW consultation and re-examination |
| Mebendazole supplementation | Proportion of children 24 months or older who needed mebendazole who received mebendazole | Number of children 24 months or older who needed mebendazole who received mebendazole | Number of children 24 months or older who needed mebendazole | Observation of HEW consultation and re-examination |
| Treatment demonstration | Proportion of children who did not need referral and were prescribed home treatment; the HEW showed the caretaker how to administer all treatments | Number of children who did not need referral and were prescribed home treatment; the HEW showed the caretaker how to administer all treatments | Number of children who did not need referral and were prescribed home treatment | Observation of HEW consultation and re-examination |
| Caretaker comprehension | Proportion of children who did not need referral and received treatment; caretaker could correctly describe how to give all treatments | Number of children who did not need referral and received treatment; caretaker could correctly describe how to give all treatments | Number of children who did not need referral and received treatment | Observation of HEW consultation and re-examination |

*(continued)*

Supplemental Appendix 1
Continued

| Indicator name | Indicator definition | Numerator | Denominator | Measurement method |
|---|---|---|---|---|
| Advising on extra fluids and continued feeding | Proportion of children with diarrhea who did not need referral whose caretaker was advised to give extra fluids and continue feeding | Number of children with diarrhea who did not need referral whose caretaker was advised to give extra fluids and continue feeding | Number of children with diarrhea who did not need referral | Observation of HEW consultation and re-examination |
| Advising on when to return immediately | Proportion of children who did not need referral whose caretaker was advised to return immediately if child cannot drink/breastfeed or gets sicker | Number of children who did not need referral whose caretaker was advised to return immediately if child cannot drink/breastfeed or gets sicker | Number of children who did not need referral | Observation of HEW consultation and re-examination |
| Advising on when to return for follow-up | Proportion of children who did not need referral whose caretaker was advised on when to return for follow-up | Number of children who did not need referral whose caretaker was advised on when to return for follow-up | Number of children who did not need referral | Observation of HEW consultation and re-examination |
| Rational use of antibiotics | Proportion of children who did not need an antibiotic who left the health post without having received an antibiotic | Number of children who did not need an antibiotic who left the health post without having received an antibiotic | Number of children who did not need an antibiotic | Observation of HEW consultation and re-examination |
| Rational use of antimalarials | Proportion of children who did not need an antimalarial who left the health post without having received an antimalarial | Number of children who did not need an antimalarial who left the health post without having received an antimalarial | Number of children who did not need an antimalarial | Observation of HEW consultation and re-examination |
| Health post opening hours | Mean number of hours that health posts were open and offering clinical services in the previous 1 week | Number of hours that health posts were open and offering clinical services in the previous 1 week | Number of health posts | HEW interview |
| Time spent by HEWs providing clinical services | Mean hours spent by HEWs providing clinical services in the previous 1 day (by health post and community) | Hours spent by HEWs providing clinical services in the previous 1 day | Number of HEWs | HEW interview |
